# Amyand's hernia a case report

**DOI:** 10.1016/j.ijscr.2021.106332

**Published:** 2021-08-24

**Authors:** Hina Khalid, Naveed Ali Khan, Munira Abdul Aziz

**Affiliations:** a Dow University of Health Sciences, Karachi 75280, Pakistan; b Dow University of Health Sciences, Karachi, Pakistan

**Keywords:** AH, Amyand's hernia, Amyand's hernia, (AH) vermiform appendix, Appendicectomy, Inguinal hernia, Hernioplasty

## Abstract

**Introduction and importance:**

Amyand's hernia (AH) is a form of inguinal hernia which is consider as very rare and this type of hernia occurred up to 1% of all inguinal hernia cases. In this type of inguinal hernia, the content of hernia sac is appendix. Most patient with AH often remains asymptomatic and diagnosed intraoperatively. The diagnosis is challenging, since needs a high index of suspicion and imaging is key. Surgery is the mainstay management. We report a case of Amyand's hernia that was managed operatively in our medium complex public institution.

**Case presentation:**

A 28 year's old man with normal body mass index (BMI) who had a history of right-side reducible linguino-scrotal swelling for 8 years, was admitted for elective right inguinal hernia repair. Two weeks back before admission, he noticed that swelling was slightly painful. Ultrasound of the abdomen reported normal findings. There was no history of abdominal pain and vomiting. Laboratory parameters were within normal limit. So, with a diagnosis of right sided partially reducible, incomplete, and indirect inguinal hernia, patient was operated for open hernia repair surgery, intra operatively we found dense adhesions within the sac, adhesions were released which revealed herniation of appendix into the inguinal canal. Appendix was mildly congested without gross evidence of inflammation. Hence, in view of noninflamed appendix, preperitoneal mesh (polypropylene) hernioplasty from Lichtenstein tension-free mesh repair was performed with appendicectomy. Postoperative period was uneventful, patient discharged at second day.

**Clinical discussion:**

Amyand's hernia is very uncommon and characterized by the presence of the appendix in the hernia sac and it is 0.4–1% of all inguinal hernia cases, literature review also showed that incidence of Amyand's hernia is very rare, whereas only 0.1% of cases complicate into acute appendicitis due to late presentation and missed diagnosis.

**Conclusion:**

Amyand's hernia (AH) makes up only a small proportion of most inguinal hernia cases, and its diagnosis is usually based on incidental finding intra-operatively. This condition may remain asymptomatic and behave like a normal inguinal hernia. Management of this type of hernia should be individualized according to appendix's inflammation stage, presence of abdominal sepsis and co-morbidity. With this approach it enables surgeons to manage more variations of Amyand's hernia. Laparoscopy for dealing Amyand's hernia is frequently diagnostic as well as therapeutic.

## 1 Background

An inguinal hernia is a protrusion of abdominal-cavity contents through the inguinal canal. Inguinal hernia sac contains any abdominal organ including small or large bowel. Amyand's hernia is a very rare and uncommon form of inguinal hernia where the vermiform appendix is present in hernia sac. The incidence of having abnormal appendix within the hernia sac varies from 0.5% to 1% [1], [2], [3], [4].

The entity of Amyand's hernia has an incidence of 1% and is complicated by acute appendicitis in 0.8to 0.13% of case, whereas only 0.1%of cases complicate into acute appendicitis [1], [2], [3], [8], [12].

Incarcerated bowel containing hernias are the leading cause of mechanical bowel obstruction. Prompt surgical intervention is indicated due to increased changes of strangulation [2], [4].

It affects both adults and kids along with also the contents of the gut sac might change from Cecum, liver, uterus, fallopian tube, omentum, or some Meckel's diverticulum along with an appendix. It received its name since the first man to report that the existence of perforated appendix was Claudius Amyand in 1735 [1], [2], [3], [4], [6].

The surgeon might encounter unusual signs, like a Vermiform appendix partially or fully found in the hernia sac, inflamed or non-inflamed or adherent into the sac walls. An inguinal hernia does not specific for age sex or group; as reported in literature, cases of Amyand's hernia found in the neonatal period for 9-2 decades, although left-sided Amyand's hernia is reported as well [1], [2], [3].

Losanoff along with the Bass-on classification System which clarifies the advocated surgical treatment selections for different kinds of Amyand's hernia. This approach will enable surgeons to recognize and manage more variations of Amyand's hernia [3] Types of Amyand's hernias and their Management [11] (Table 1).

**Table 1:** Losanoff and Basson's classification [11].

Classification	Description	Surgical management
Type 1	Normal appendix within an inguinal hernia	Hernia reduction, mesh repairs, appendectomy in young patients
Type 2	Acute appendicitis within hernia, no abdominal sepsis	Appendectomy through hernia primary repair of Hernia, no mesh
Type 3	Acute appendicitis within an inguinal hernia, abdominal wall, or peritoneal sepsis	Laparotomy, appendectomy, primary repair of hernia, no mesh
Type 4	Acute appendicitis within an inguinal hernia, related or unrelated abdominal pathology	Manage as type 1 to 3 hernia investigate or treat second condition as appropriate

## 2 Case presentation

### 2.1 Patient information

A 28-year-old male with normal BMI presented to a surgical department clinic with complaint of a pain and swelling in the right inguinal region for the past two weeks initially it was small and gradually increase in size and associated with pain. This swelling was present for the last 8 years causing no symptoms, so the patient did not seek any medical advice for it. There was no history of abdominal pain and vomiting. The patient is an engineer by profession, suffering from Hepatitis C, for which he took treatment.

### 2.2 Physical examination

On examination, there was an indirect right inguinal hernia incomplete, and reducible type with positive cough impulse.

### 2.3 Diagnostic assessment

Patient was diagnosed clinically as indirect inguinal hernia. Hematological workup was within normal limits.

### 2.4 Intervention

A Preoperative diagnosis of right inguinal hernia was made and was planned for hernia mesh repair, during surgery under spinal anesthesia, the hernia sac was found to contain an appendix. The appendix was slightly congested, not inflamed but there were dense adhesions within the sac and so adhesiolysis and appendectomy along with excision of the sac and Lichtenstein mesh hernioplasty was done. The postoperative period was uneventful. The patient postoperatively received fluid therapy, Oral fluids were administrated after 6 h along with soft diet and was discharged on postoperative day 2.

### 2.5 Follow-up and outcome

**Fig. 1:**
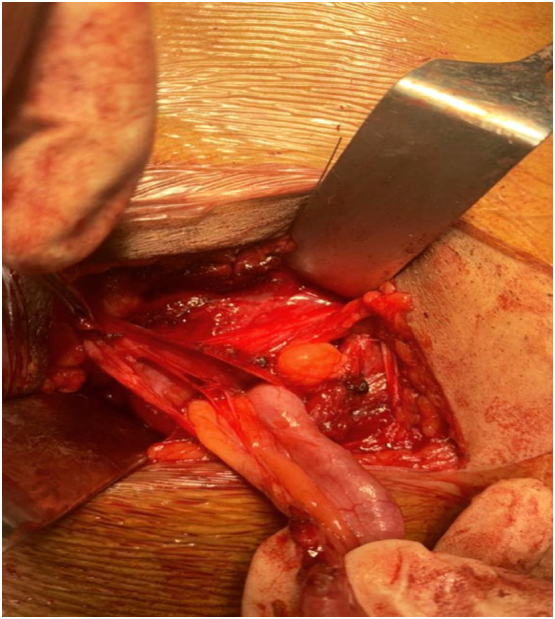
Adhesions around deep inguinal ring with herniation of appendix.

**Fig. 2:**
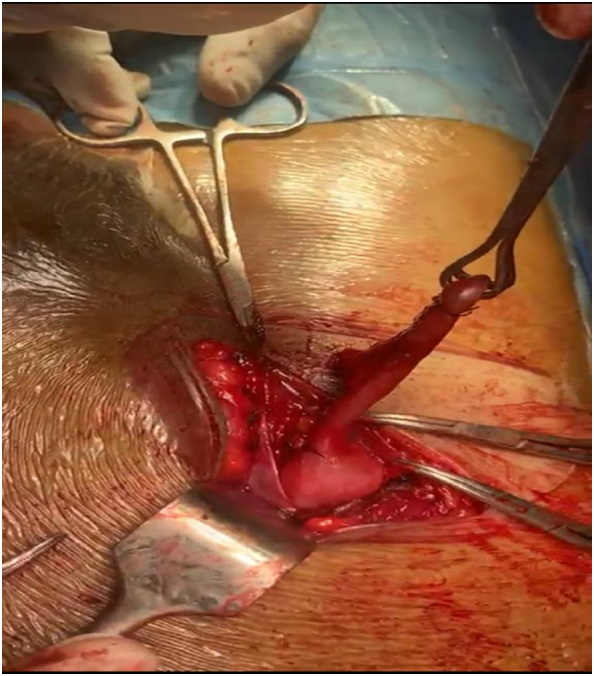
Lateral view of appendix within right inguinal hernia sac.

**Fig. 3:**
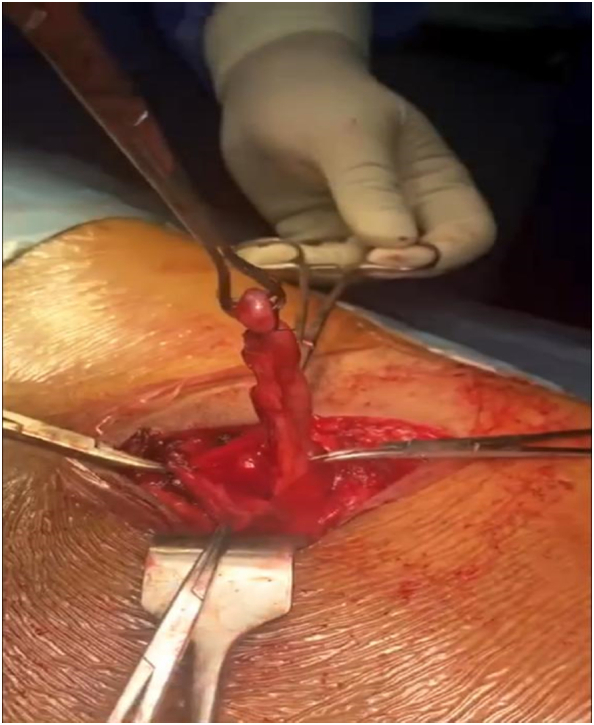
Amyand's hernia, appendix in hernia sac, Appendicectomy being performed after ligation of mesoappendix in the hernia sac.

The patient was discharged on second post-operative day with complications on oral anti-biotics and followed up after one week of surgery (Fig. 1, Fig. 2, Fig. 3).

## 3 Discussion

Amyand's hernia (AH), the disease has been originally clarified and handled in 1735 in Claudius [1], [2], [3]. Amyand Losanoff and Basson indicate Amyand's hernia may be dealt with reduction or appendectomy, based on co-morbidities, Amyand's hernia accounts for 0.1 percentage of instances of all appendicitis [1], [3], [4], [5], [6]. Appendectomy with simultaneous hernioplasty was completed prophylactically for potential future complication that may lead to appendicitis [11].

The Choice to keep or remove the appendix relies upon the individual's era, endurance, and hazards of expanding acute appendicitis. The young people include a much greater chance of afflicted by acute appendicitis in contrast for this middle era or elderly men and women [12]. Amyand's hernia is also a rare and hard to diagnose disease, being often occasionally discovered intraoperatively [2], [3], [4].

In terms of inguinal hernia detection, the initial use of ultrasound possibly followed by CT, represents a sensitive and cost-effective progression for the evaluation of the patient with a clinical history suggestive of a hernia, enables the possibility of differentiating affiliated intra-abdominal organs [4], [6].

Therefore, an appendectomy had to be performed to enable structural reduction of the hernia despite the absence of visible signs of acute appendicitis [4].

Considering ultrasound imaging in a large-sized inguinal hernia even if it is reducible may be beneficial in reducing the complications. Early analytical imaging use may confound an instant pre-operative identification of AH. Right-sided Amyand's hernias occur more often than left due to the anatomical location of the appendix on the right. Left-sided Amyand's hernias are very rare [3]. Laparoscopic repair has also been described in pediatric age group [7].

The mainstay management is the open surgery, but in recent years the laparoscopy approach is summing cases; giving benefits of shorter hospital stay, faster recovery, less postoperative pain, among others [12]. While CT scan may help in making a preoperative diagnosis in those cases presented with an acute abdomen the diagnosis of Amyand hernia is usually made intraoperatively [11].

The underlying mechanisms that cause acute appendicitis within an Amyand's hernia include decrease blood supply of the appendix due to adhesions that may cause non-reducibility of the hernia and compression in the external ring originating from increases in intra-abdominal pressure [10]. These factors lead to recurrent inflammation and bacterial overgrowth. The intraoperative manipulations that can by themselves trigger an inflammatory process.

The Losanoff and Basson's classification presented above offer satisfactory guidance system for management of Amyand's hernia [9]. A normal looking appendix in the hernial sac does not always require appendectomy. Appendectomy adds the risk of infection to an otherwise clean procedure. Whether to remove or leave behind a normal appendix is a clinical dilemma because no evidence-based information exists. The decision should be based on common sense, taking into account the patient's age, life expectancy, life-long risk of developing acute appendicitis, and the size and overall anatomy of the appendix.

Definitive preoperative diagnosis poses a challenge due to indistinct clinical signs and symptoms. We report a case that was managed by open surgery in our medium complex public institution with review of the literature. This case report is in line with SCARE 2021 guidelines.

## 4 Conclusion

Amyand's hernia is a rare presentation of inguinal hernias. In the clinical setting of an incarcerated complicated or strangulated inguinal hernia, the initial approach should consider imaging studies; USG or CT can guide the surgical plan, and enables the possibility of identifying involved intra-abdominal organs. Literature review recommends reducing the hernia content and perform no tension hernia repair, In the cases where an inflamed, suppurative or perforated appendicitis were encountered, no prosthetics material should be used because of the increased risk of surgical site infection.in case of clinical suspicion of Amyand hernia is diagnostic laparoscopy which is useful approach in all forms of incarcerated hernias to assess contents and avoid unnecessary laparotomy We are still conservative about the application of mesh in hernia sac with acute appendicitis, which requires additional large-scale study to determine whether mesh repair will increase the risk of infection or not.

## Ethical approval

No ethical approval is required. Our institution does not demand ethical approval for case reports, as these projects are not investigational.

## Financial support and sponsorship

None declared.

## CRediT authorship contribution statement

Hina Khalid, conceptualization, data curation, writing – original draft.

Naveed Ali khan, review & editing.

Munira Abdul Aziz, review & editing.

## Guarantor

Hina Khalid

## Consent for publications

Written informed consent was obtained from the patient.

## Provenance and peer review

Not commissioned, externally peer reviewed.

## Declaration of competing interest

There are no conflicts of interest.
